# A machine learning algorithm to analyse the effects of vaccination on COVID-19 mortality

**DOI:** 10.1017/S0950268822001418

**Published:** 2022-09-12

**Authors:** Cosimo Magazzino, Marco Mele, Mario Coccia

**Affiliations:** 1 Department of Political Sciences, Roma Tre University, Roma, Italy; 2 ‘Niccolò Cusano’ University, Roma, Italy; 3 CNR-National Research Council of Italy, Roma, Italy

**Keywords:** Clinical decision support system, COVID-19 vaccines, crisis management, infectious diseases, machine learning, public health, vaccination campaign

## Abstract

The coronavirus disease 2019 (COVID-19), with new variants, continues to be a constant pandemic threat that is generating socio-economic and health issues in manifold countries. The principal goal of this study is to develop a machine learning experiment to assess the effects of vaccination on the fatality rate of the COVID-19 pandemic. Data from 192 countries are analysed to explain the phenomena under study. This new algorithm selected two targets: the number of deaths and the fatality rate. Results suggest that, based on the respective vaccination plan, the turnout in the participation in the vaccination campaign, and the doses administered, countries under study suddenly have a reduction in the fatality rate of COVID-19 precisely at the point where the cut effect is generated in the neural network. This result is significant for the international scientific community. It would demonstrate the effective impact of the vaccination campaign on the fatality rate of COVID-19, whatever the country considered. In fact, once the vaccination has started (for vaccines that require a booster, we refer to at least the first dose), the antibody response of people seems to prevent the probability of death related to COVID-19. In short, at a certain point, the fatality rate collapses with increasing doses administered. All these results here can help decisions of policymakers to prepare optimal strategies, based on effective vaccination plans, to lessen the negative effects of the COVID-19 pandemic crisis in socioeconomic and health systems.

## Introduction

The coronavirus disease 2019 (COVID-19) is an infectious illness caused by the novel severe acute respiratory syndrome coronavirus 2, which appeared in late 2019 [1–4]. COVID-19 is still circulating in 2022 with mutations of the novel coronavirus that generate new variants of concern^1^ driving continuous infections and deaths in manifold countries [5–7]. Seligman *et al*. [8] show some characteristics of people that are significantly associated with COVID-19 mortality, such as mean age of 71.6 years, non-white race/ethnicity, income below the median, and less than a high school level of education. High numbers of COVID-19-related infected individuals and deaths worldwide have supported the development of different types of vaccines from 2020 based on viral vector, protein subunit and nucleic acid [9–12]. In the presence of the COVID-19 pandemic crisis, the investigation of vaccination plans is a crucial aspect to determine how the novel infectious disease can be controlled and/or eradicated in the population [13]. Vaccination has the potential effect to reduce the diffusion of COVID-19, relaxing non-pharmaceutical measures, and maintain low basic reproduction number; nevertheless, an important point to clarify is the optimal strategy of administering the vaccines during the pandemic evolution wave, to reduce negative effects in society [14]. Akamatsu *et al*. [15] argue that the vital role of governments is directed to implement an efficient campaign of vaccination to substantially reduce infections and mortality in society and avoid the collapse of the healthcare system. Aldila *et al*. [13] maintain that higher levels of vaccination rate can eradicate COVID-19 in the population by approaching herd immunity to protect vulnerable individuals [16–19]. Rosen *et al*. [20] describe socio-economic and organisational factors associated with the success of the vaccination campaign in Israel as well as they show some aspects of misinformation that can reduce the effectiveness of a fruitful vaccination plan over time [21]. In this context, a vital problem in the current COVID-19 pandemic crisis is the effective level of vaccination that supports a drastic reduction of infected individuals and deaths.

The present study confronts this problem by developing a machine learning (ML) algorithm to empirically assess the effects of vaccination on deaths and the fatality rate of the COVID-19 pandemic. The application of ML approaches can provide a new perspective to support appropriate policy responses of policymakers directed contrast the outbreaks of new variants of COVID-19 and similar infectious diseases in the future [22–29].

The goal of this paper is to show the role of ML experiments as one of the significant methods in the research arena for predicting and assessing the effects of vaccination on health and improving crisis management of the COVID-19 pandemic. The results can suggest best practices of optimisation in the vaccination strategy to guide effective and timely policy responses for combatting the novel virus and constraining the negative effects of the COVID-19 pandemic crisis and future epidemics of similar infectious diseases in society. The findings of this study could be also of benefit to countries as they grapple to plan their vaccine plans for the COVID-19 pandemic crisis to minimise the negative effects of the pandemic crisis on the environment and socio-economic systems. This study is part of a large body of research projects directed to explain the drivers of transmission dynamics of COVID-19 and design effective policy responses to cope with and/or prevent pandemic threats [30–36].

The rest of the paper is organised as follows: Section ‘Literature review on artificial intelligence (AI) studies on COVID-19 vaccines effects’ presents the literature on studies that employed AI techniques to explore the effects of COVID-19 vaccines. In Section ‘Materials and methods’ we describe the methodologies applied and the dataset. Section ‘Empirical results’ presents the empirical findings. Finally, Section ‘Conclusions and policy implications’ concludes and gives the limitations of the study together with some policy recommendations.

## Literature review on artificial intelligence studies on COVID-19 vaccines effects

Several studies analysed the effects of vaccination campaigns on COVID-19 diffusion through AI approaches.

Several papers have been devoted in the more recent years to provide surveys on literature reviews or systematic reviews regarding the COVID-19 pandemic. Abd-Alrazaq *et al*. [37] elaborated an extensive bibliometric analysis to offer a comprehensive overview of the literature on COVID-19. Lv *et al*. [38] showed an extensive survey on the application of AI and ML to defeat the COVID-19 pandemic. Wang *et al*. [39] performed a systematic review of the application of AI techniques related to COVID-19. The results showed that AI obtained high performance in diagnosis, prognosis evaluation, epidemic prediction and drug discovery for the virus. De Felice and Polimeni [40] conducted a bibliometric analysis using an ML bibliometric methodology. Zyoud and Al-Jabi [41] run a bibliometric analysis to get a plausible scenario of the COVID-19 pandemic crisis.

Lincoln *et al*. [42] investigated the willingness to get a vaccine over a sample of five advanced countries using an ML algorithm. Vaccination conspiracy belief was found to represent the most relevant predictor.

Many AI applications analysed the public sentiments towards vaccines using social media data. Xue *et al*. [43] analysed 4 million Twitter messages regarding the COVID-19 pandemic. The Latent Dirichlet Allocation (LDA) findings revealed the anticipation that measures can be taken as the dominant sentiment for the virus spread. Liew and Lee [44] used social media data to understand public sentiments about COVID-19 vaccines through an unsupervised ML approach (structural topic modelling). They found that tweets with negative feelings were about emotional reactions. Lyu *et al*. [45] identified the main topics present in the public discussion of the COVID-19 vaccines on social media by applying an LDA for topic modelling. The emotion analysis showed the main emotion was trust, followed by anticipation, fear and sadness. Kwok *et al*. [46] built an LDA topic model to reveal topics and sentiments on COVID-19 vaccination on Twitter in Australia. Three topics emerge: attitudes toward COVID-19 and its vaccination; advocating infection control measures against COVID-19; misconceptions and complaints about COVID-19 control. Lian *et al*. [47] developed an ML and Natural Language Processing (NLP) approach to discover COVID-19 vaccine adverse events using data from Twitter. The results show that the four most populous states in the US (California, Texas, Florida and New York) recorded the most discussions on Twitter, highlighting a strong correlation between Twitter discussions and vaccination campaigns.

A different strand of literature used AI approaches to inspect the effect of contact tracing apps. Cresswell *et al*. [48] explored public perceptions of COVID-19 contact tracing apps in the UK using a deep learning (DL) approach, revealing 76% positive sentiments. Hussain *et al*. [49] analysed public sentiments on social media towards COVID-19 vaccines in the UK and the US by applying NLP and DL-based techniques. The results show a majority of the overall averaged positive sentiments in both countries. Weiß *et al*. [50] developed an item set to monitor nationally issued COVID-19 contact tracing apps using an Open-Source Intelligence approach. Empirical findings highlighted differences among the countries in the sample.

Bagabir *et al*. [51] tried to highlight the advantages of AI applications to identify the genomic sequences as well as the development of drugs and vaccines for COVID-19. Monteleone *et al*. [52] investigated the impact of AI on drug repurposing of therapies for the treatment of COVID-19.

## Materials and methods

### Source and sample

The sample of this study is based on *N* = 192 countries worldwide. The period under study is from March to May 2021, using data on vaccines, confirmed cases, and the fatality rate of COVID-19. The list of countries under study is in the Appendix.

### Measures

– Doses of vaccines administered × 100 inhabitants on 15 March 2021 with *N* = 114 countries; on 14 April 2021 with *N* = 154 countries and on 26 April 2021 with *N* = 190 countries. The number of samples tends to increase over time with the diffusion of vaccines across countries worldwide. Doses of vaccines refer to the total number of vaccine doses, considering that an additional dose may be obtained from each vial (e.g. six doses for Pfizer BioNTech^®^ Comirnaty), whereas the number of doses administered refers to any individual receiving any dose of the vaccine [53, 54]. The data here considers all types of COVID-19 vaccines used in different countries, i.e. vaccines by Johnson & Johnson, Oxford/AstraZeneca, Pfizer/BioNTech, Sinopharm/Beijing, Sinovac, Sputnik V and Moderna [55]. Of course, every country has been using a different combination of these COVID-19 vaccines to protect the population [56–58]. Source of data: Our World in Data [59].
– Number of COVID-19 infected individuals (%) is measured with confirmed cases of COVID-19 divided by the population of countries under study on 20 March 2021 (*N* = 192 countries), 25 April 2021 (*N* = 192) and 19 May 2021 (*N* = 216 countries). Source of data: Johns Hopkins Center for System Science and Engineering [5].
– Number of COVID-19 deaths is measured with case fatality rate (%) given by deaths on 25 April 2021 divided by the total infected individuals in each country. Source of data: Johns Hopkins Center for System Science and Engineering [5].

### Data analysis procedure

Once we have collected the data and postulated it according to a rigid regime consistent with the ML hypotheses framework, we search for the best algorithm to optimise the dataset. The data computerisation process aims to train the machine to process results without any further intervention by the operator. In other words, the initial computer programming instructions are only necessary for it to carry out an autonomous but at the same time specific behaviour in the processing of data and results. Among the numerous approaches that an ML process can use, we have chosen to use the Artificial Neural Networks (ANNs). The empirical analyses are conducted in Oryx, and for the analysis of the neural network (NN) AD-Designer is used. The choice to use an NN process coincides with the assumption of discrimination. In general, we can say that a discriminant function is a function that receives the input data as input and whose output represents the classification of such data. Furthermore, the discriminant function can be generalised by transforming the result through a non-linear function called the activation function.

So, our experiment began with the activation function. It is responsible for updating the status of the *i*-th neuron as it transitions from one moment to the next:1
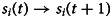


Thus, the stimulation input of the neuron *i* of the *σ*-layer with respect to our *n*-layer feed-forward network was given by the potential 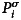:2
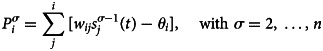


The threshold *θ*_*i*_ has been dropped, while an emulated input *s_k_* = 1 is added, to which we have assigned the relative connection weight:3
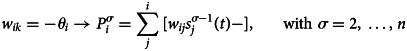


The subsequent state of the neuron *i*, *s_i_*(*t* + 1), is computed by an appropriate function of the potential *P_i_*, called the activation or transfer function:4
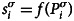


The function *f* can take various forms, but the one used here is the hyperbolic function of the type: sine, cosine and secant.

Then, the training phase is activated. In the training phase, the NN starts from an initial state characterised by the assignment of arbitrary values for the synaptic weights. It dynamically evolves towards a final equilibrium state corresponding to the learning of the problem in question. In the context of the connectionist paradigm, learning assumes a fundamental importance: it is generally not possible to fix in advance the weights of the connections between neurons, depending on the task that the NN has to perform. These weights must be learned and the NN must behave like an adaptive system. The learning phase is very long. It requires the presence of a set of input vector values with known output (supervised training). This dataset forms the set of examples (training set) that the NN must learn and emulate. The adaptation of the weights of the links was done by using iterative procedures; here retroactive procedures are chosen. They consist in comparing the values computed by the network with those to be emulated. Then, the weights of the connections are modified to minimise the difference in this comparison. This procedure is repeated for each example, i.e. for each vector of the training set. For the NN to reach its final structure, a single training cycle is not sufficient: many iterations are needed for this experiment to reach a satisfactory approximation. Our algorithm for the backpropagation of faults finds a place in numerous applications due to its generalisation ability. However, it has the limitation that it is very slow in the learning process. It requires many cycles before it reaches a sufficiently small global error. However, we have accelerated the algorithm with the following modification of the weight update law:5
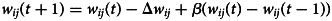


The error function obtained is considered as a function of the energy of the system. The state of minimum energy corresponds to the equilibrium state of the same. The error function, when the transfer (or activation) function is linear, is represented by a hyper paraboloidal surface with an absolute minimum. Learning corresponds to reaching the configuration with minimum energy and is guaranteed since the hyper paraboloid has only an absolute minimum. When the activation function is non-linear but monotonic, like the sigmoid function, it is represented by a deformed hyper paraboloid characterised by the presence of relative minima. Thus, once the best estimation model is defined and its activation function is specified, we can develop a prediction process capable of estimating the number of deaths and the mortality rate with respect to a set of inputs attributable to seven variables: Total Cases, Cases/Population, Vaccine Dose (April), Vaccine Dose per 100 Population (April), Vaccine Dose (15–21 March), Vaccine Dose per 100 Population (15–21 March), Total Vaccination (15–21 March). Since the algorithm needs to read a large dataset with numerous observations, we extended the dataset using various mathematical transformations. In particular, for each variable, we created its logarithm and the first difference transforms. We then trained the machine to generate an NN based on a combination of 6 nodes distributed according to the following scheme: 12-10-7-5-7 and 2 output layers. Therefore, the neural series analysis process is defined through the following scenario: Scaling Layer methods: Mean Standard Deviation; Unscaling layer: Minimum-Maximum; Bounding layer: no apply bounding layer; Maximum iterations: 100 000; Maximum time (hh: mm): 01:00; Maximum failures: 1.

## Empirical results

**Fig. 1.**
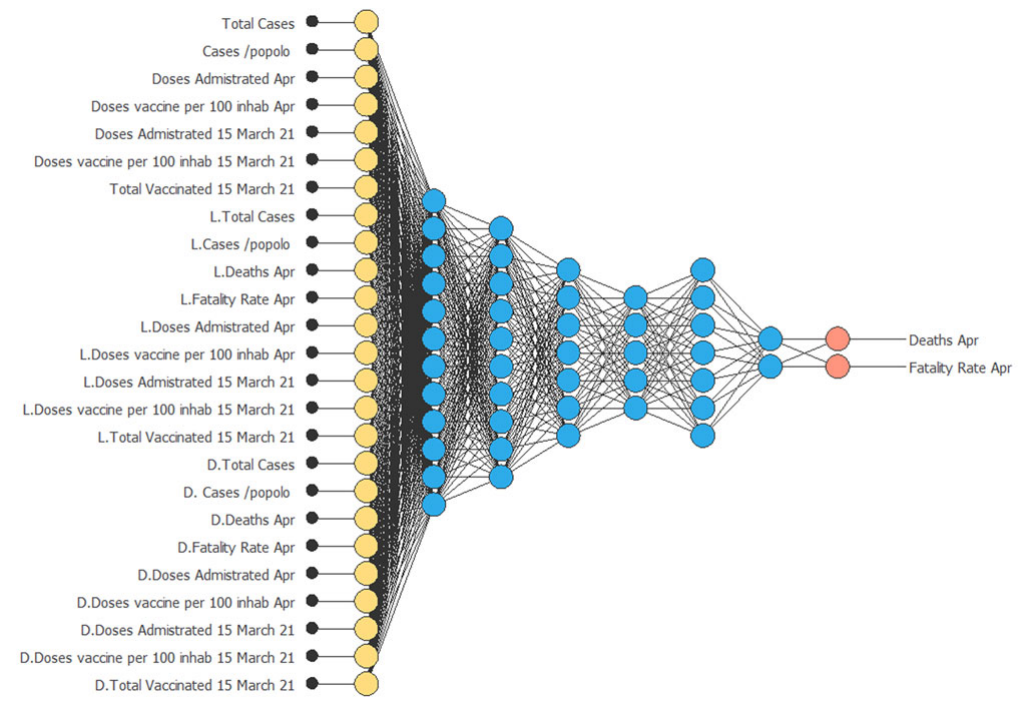
Constructed ANN. *Source*: our elaborations in Oryx.

After demonstrating the mathematical results of the newly constructed algorithm, in this section we show the results obtained with the NN. The dataset includes *n* + 1 countries (i.e. 192) distributed according to the seven variables (plus their mathematical transformations): The architecture of the derived NN is shown in Figure 1. We can see that the NN is of multivariate type. Two targets attributable to the variables deaths (April) and mortality rate (April) were isolated.

**Fig. 2.**
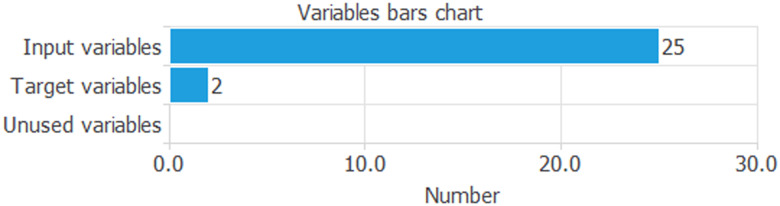
Variables bars chart. *Source*: our elaborations in Oryx.

Now, we analyse the ANNs results. In total, the experiment consists of 25 inputs and 2 targets (Fig. 2).

**Fig. 3.**
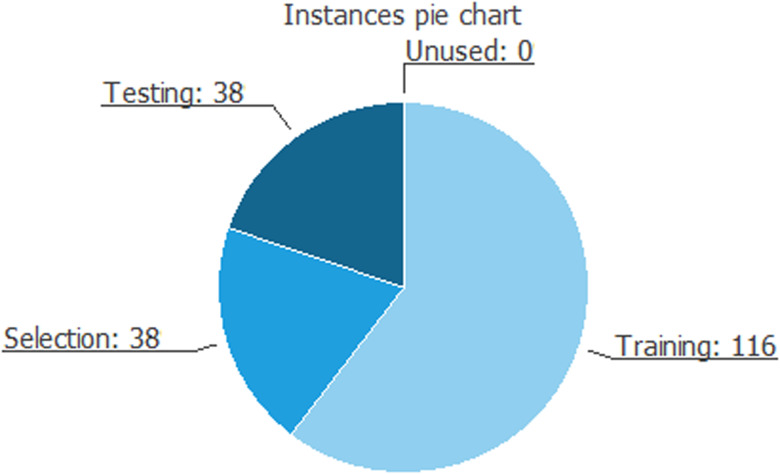
Instances pie chart. *Source*: our elaborations in Oryx.

Through the pie chart in Figure 3, we can observe in detail the use of all instances in the dataset.

The total number of instances is 192. The number of training instances is 116 (60.4%), the number of selection instances is 38 (19.8%), the number of testing instances is 38 (19.8%), and the number of unused instances is 0 (0%). These results can be explained as follows: the training instances designed the best process for the neural algorithm. It has a design accepted 60 times out of 100 and its value is higher than another algorithm (selection instance) and the same value as the testing instance.

**Fig. 4.**
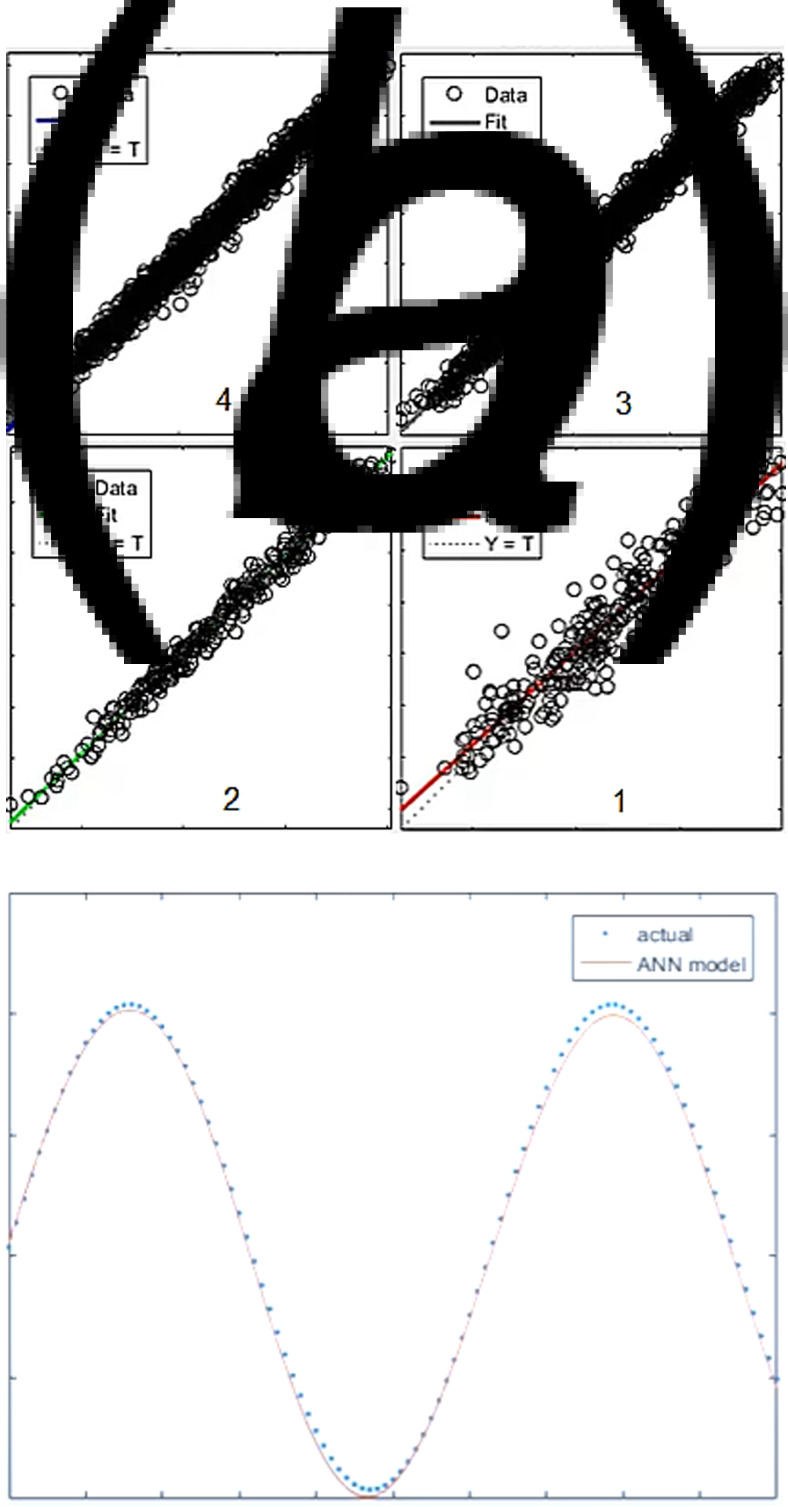
Fit model process. *Source*: our elaborations in Oryx.

Before analysing the results obtained on the target variables, we observe the behaviour of the data within our NN structure. We interrogate the algorithm to illustrate how the real signals among the numerous nodes are compatible with the predicted ones. The underlying hypothesis is that the NN prediction process coherently reflects the environment and thus the reality of the data with respect to an unsupervised approach. The results are shown in Figure 4.

The algorithm starts from a signal optimisation process that one can imagine coinciding with a dated time 1. Subsequently, as the signal (and therefore the data distribution) is different from the respective weights between the nodes, the algorithm begins to choose the best signal optimisation process. This procedure is an automatic learning process resulting from 1796 strings of commands that leave the machine in a situation of quasi-controlled autonomy. Therefore, the figures named with numbers from 2 to 4 result from the signal optimisation process. In fact, we can observe how at time 4 (top left panel) the predicted data coincide perfectly with the predicted ones. The methodological summary of graphs 1–4 is represented in the last figure below. Thus, it is easy to see how the NN model can perfectly predict data coming from the real world. Once we have observed that our NN is able to generate highly reliable predictive values, we estimate which input might explain the desired targets. To this end, according to Mele *et al*. [4], we use the ‘Plot Directional Output’ function. In other words, we implement the new algorithm through what is commonly called the ‘cut effect’ of the NN. Hence, the following commands into the algorithm have been introduced:

*Core commands:*

**Table:** 

subplot (x,y,z)
hold on
plot (data1(1, f:f + length));
plot (data2(1, f:f + length));
plot (data3(1, f:f + length));
plot (data4(1, f:f + length));
plot (data4 + n(1, f:f + length));
hold off
%%computation
angle(2, f: f + length), detal (3, f:f + length)….frequency);
f2 )fft (sig1);
f3 )fft (sig2);
fn )fft (sig n);
[‥, p1] = max(abs(f1)
[‥, p2] = max(abs(f2)
[‥, p3] = max(abs(f3)
bearing = ata((t3-t2)
end

**Fig. 5.**
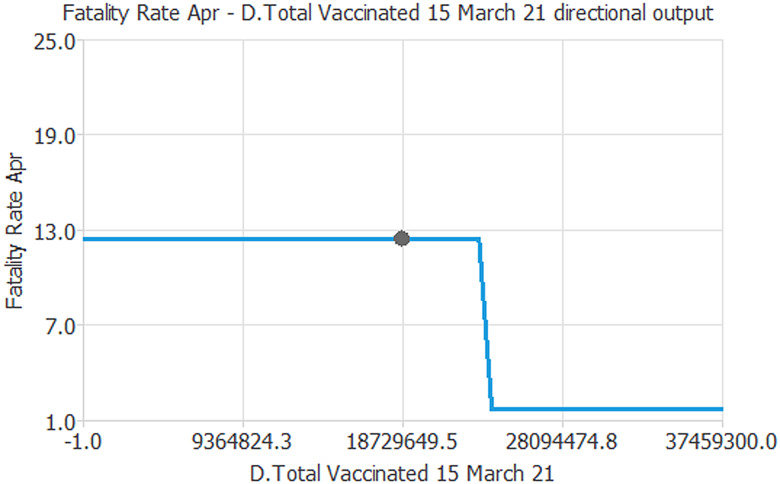
‘Cut Effect’ based on a new ML algorithm. *Source*: our elaborations in Oryx and AD-Designer 2021.

The results of the ‘cut effect’ analysis in the NN are illustrated in Figure 5. In general, the ‘Plot Directional Output’ function analyses numerous combinations of inputs on the outputs, automatically choosing the one that can influence the most network signal process.

Figure 5 shows the vital result of the experiment here. The cut-off signal in the transmission of the nodes generated the following propagation: from the input ‘D.Total Vaccinated 15 March 21’ to the target ‘Fatality Rate April’ it has been identified at point 12.8 regarding the ordinate *y*-axis (target) and level 26079511.3 concerning the abscissa *x*-axis (input). This result requires an interpretation of the data that has been cut during the signal propagation. In particular, considering 192 countries under study here, the respective vaccination plan, the turnout in the participation in the vaccination campaign, and the doses administered, these countries suddenly saw a reduction in the Case Fatality Rate of COVID-19 precisely at the point where the cut effect was generated in our NN.

**Fig. 6.**
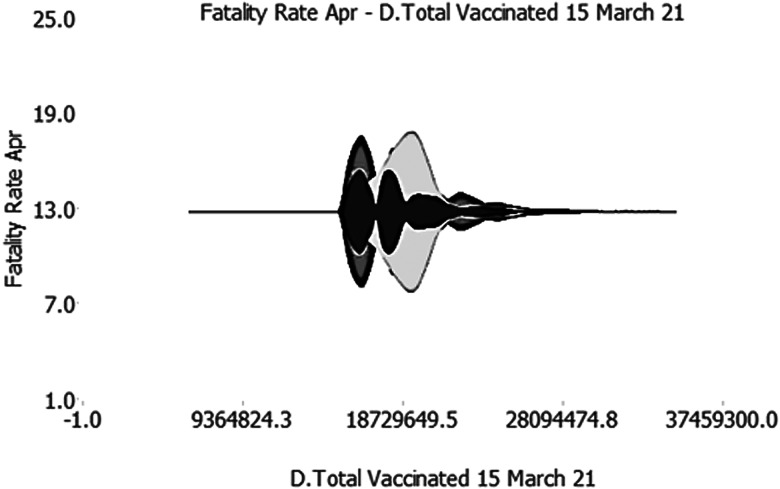
Signal amplification process. *Source*: our elaborations in Oryx and AD-Designer 2021.

This result is significant for the international scientific community. It would demonstrate the effective impact of the vaccination campaign on the Case Fatality Rate from COVID-19, whatever the country considered. Indeed, once the vaccination has started (for vaccines that require a booster, we refer to at least the first dose) the antibody response seems to prevent the probability of death from COVID-19. The graph of the cut effect is clear: at a certain point, the Case Fatality Rate collapses with increasing doses administered. To be able to understand the delay effect in the activation of neutralizing antibodies (14 days of the activation of IgG antibodies), we have used a signal amplification process (Fig. 6).

According to the Qiagen experiment (Valencia 2007) and the Siemens approach (Tarrytown 2006), our algorithm simulates the signal amplification process used in virology. This technique was introduced to overcome the limitation of polymerase chain reaction tests. However, unlike the signal test in virology, since we did not use the enzyme rotation techniques, we simulated that the neural weights can be amplified in *n* + 1 cycles, to make the underlying factors more visible.

It was built through a new algorithm implementation that generated the cutting effect. In other words, through this analysis, we have interrogated the machine to illustrate time delays in the propagation of the signal before the algorithm detects the cut effect. Thus, with this methodology, we have created a hypothetical premise to grasp the 14 days of delay necessary for the human body to produce immunity. The result is really interesting, as we can see that the signal undergoes a significant amplification even before the cut relative to Figure 5. Therefore, this observation allows us to limit the gap between those who start vaccination (but immediately produce the IgM) and colours who within and beyond 14 days acquire protective immunity from COVID-19.

**Fig. 7.**
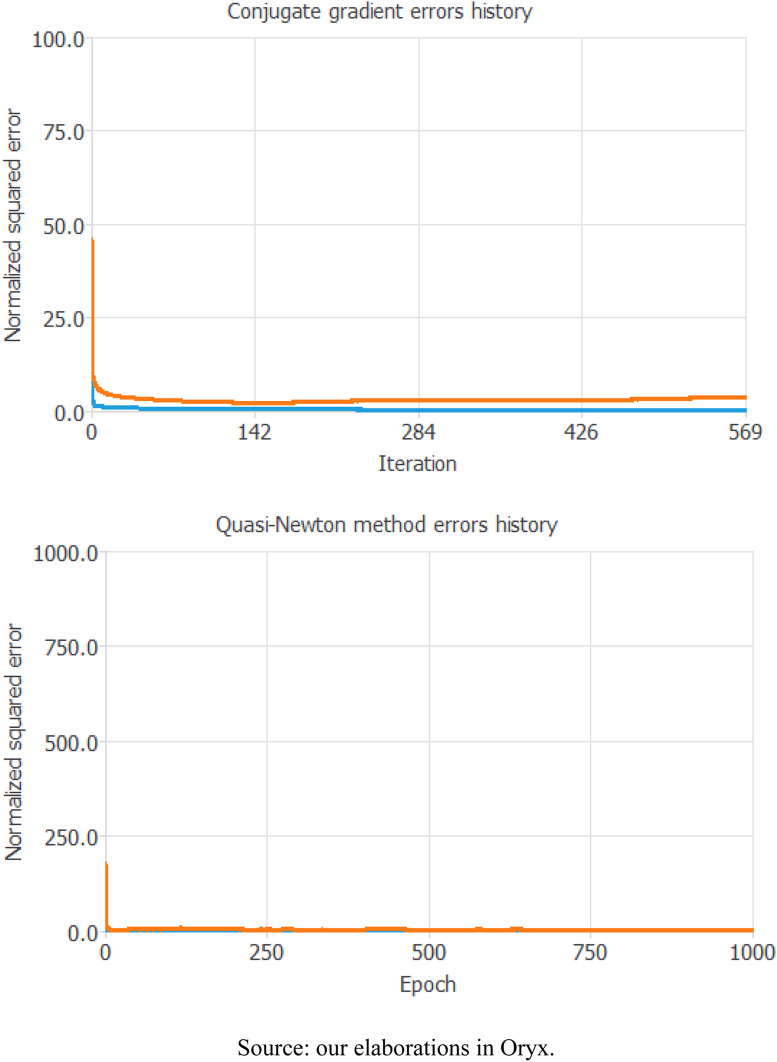
ML diagnostic tests. *Source*: our elaborations in Oryx.

Finally, to check for errors in the prediction process, we test our model through two different tools: the Conjugate gradient errors history and the Quasi-Newton method (Fig. 7).

Both methods follow the belief that the best training strategy is the one that allows the best possible loss of information as interactions or epochs grow. The conjugate gradient is used for training. In this algorithm, the search is performed with conjugate directions, which generally leads to faster convergence than the gradient descent directions. The initial value of the training error (orange line) is 49.6111, while the final value after 569 iterations is 0.279563. The initial value of the selection error (blue line) is 4.2048 and the final value after 5 ITE is 0.000243. The analysis of this test confirms that the error in our prediction the error decreases as the iterations increase, and this result confirms the goodness of the selected model.

Instead, the Quasi-Newton method computes an approximation to the inverse Hessian at each iteration of the algorithm using only the gradient information. The blue line represents the training error, and the orange line represents the selection error. The initial value of the training error is 0.065332 and the final value after 1000 epochs is 0.0005. The initial value of the selection error is 112.0454 and the final value after 1000 epochs is 0.0004. This analysis, which confirms the previous one, also shows that the whole selection and training process of our NN has almost no error as the epoch increases.

## Conclusions and policy implications

COVID-19 and future epidemics of novel influenza viruses pose more and more a serious threat to the security and public health of nations [1, 6, 30, 31, 32, 34, 35]. The global response to the COVID-19 pandemic has pushed the research for detecting factors and aspects associated with a rapid pandemic response in several areas, including vaccine development, distribution, allocation and administration. This study suggests efficient strategies of vaccination for reducing the impact of the novel viral agent that might not disappear in the short-term because of new variants [60]. We demonstrated, through a new ML algorithm, that the vaccination campaign significantly reduced the negative effects of the COVID-19 pandemic, with a sharp decrease in the fatality rate. In particular, results suggest that, based on the respective vaccination plan, the turnout in the participation in the vaccination campaign, and the doses administered, countries under study suddenly have a reduction in the fatality rate of COVID-19 precisely at the point where the cut effect is generated in the NN. This result is significant for the international scientific community. These results here can help policymakers to design satisfying goals to cope with current infectious diseases with effective vaccination strategies to prevent future outbreaks of new variants of COVID-19 and similar infectious diseases in the future.

Although this study has provided some interesting results, that are of course tentative, it has several limitations. First, a limitation of the study is the lack of data about doses administered and total vaccinations in several countries, mainly in the spring season of the year 2021, and also the difficulty of production and distribution of COVID-19 vaccines worldwide; moreover, country-specific health norms may affect the gathering and transmission of data, such that unreported doses of vaccines and deaths in manifold countries may be present in the database under study here. Antony *et al*. [61] explained that the similarity in presentation between COVID-19 and influenza can have generated underreported data across countries. Second, not all the possible confounding factors that affect the efficacy of vaccinations are taken into consideration (such as non-pharmaceutical measures applied, health expenditures, numbers of ICUs, equipment of medical ventilators in hospitals, hesitancy to vaccination, etc.) and in the future, they deserve to be controlled for reinforcing results here. Third, the lack of integration of data with the age of vaccinated people among countries (the priority given in many countries to elderly subjects, with a more compromised immune system) may have influenced the results of infected individuals and deaths across countries. Fourth, new variants of COVID-19 may reduce the effectiveness of current vaccines and vaccination campaigns and these aspects have to be considered in the future development of this study. Thus, generalizing the results of this research should be done with caution. Despite these limitations, the results presented here clearly illustrate the critical level of vaccination rollout that has significantly reduced the negative effects of the COVID-19 pandemic in terms of a sharp decrease in the case of fatality rates across countries. These findings can better support the strategies for prevention and/or reduction of negative effects of the pandemic crisis in society [30, 33]. Future research should consider new data when available, and when possible, also examine time series of variables within countries to explain more dynamic relations of the phenomena and relationships under study here over time and space. In fact, as the vaccination campaign progresses around the world, it would be interesting to repeat the analysis conducted here to check if the results we have presented are confirmed through a larger sample. Moreover, the empirical analysis may be conducted using a different AI algorithm or a completely diverse approach [27].

To conclude, there is a need for much more detailed research on these topics and this study encourages further investigations for supporting optimal strategies of vaccination plans, using lessons learned from COVID-19, also considering the interaction between the evolution of pandemics with new variants and vaccination campaigns, and different factors between countries that are not only parameters related to medicine but also to public governance that can clarify results and improve the preparedness of countries to face next pandemic crisis and control negative impact on public health, economy and society.

## Data Availability

The data that support the findings of this study are available on request from the authors.

## Footnotes

1 SARS-CoV-2 variants of concern until July 2022: Omicron BA.1, BA.2, BA.4, and BA.5 [59].

## Supplementary material

For supplementary material accompanying this paper visit https://doi.org/10.1017/S0950268822001418. click here to view supplementary material

## Appendix

A. Core Mathematical Results

The mathematical expression represented by the NN is written below. It takes the inputs Total Cases, Cases/popolo, Doses Admistrated April, Doses vaccine per 100 inhab April, Doses Admistrated 15 March 21, Doses vaccine per 100 inhab 15 March 21, Total Vaccinated 15 March 21, L.Total Cases, L.Cases/popolo, L.Deaths April, L.Fatality Rate April, L.Doses Admistrated April, L.Doses vaccine per 100 inhab April, L.Doses Admistrated 15 March 21, L.Doses vaccine per 100 inhab 15 March 21, L.Total Vaccinated 15 March 21, D.Total Cases, D. Cases/popolo, D.Deaths April, D.Fatality Rate April, D.Doses Admistrated April, D.Doses vaccine per 100 inhab April, D.Doses Admistrated 15 March 21, D.Doses vaccine per 100 inhab 15 March 21, D.Total Vaccinated 15 March 21 to produce the outputs Deaths April, Fatality Rate April. For function regression problems, the information is propagated in a feed-forward fashion through the scaling layer, the perceptron layers and the unscaling layer.

1. Construction of the activation function

scaled_TotalCases = (TotalCases−597 159)/1 804 230;

scaled_Cases_popolo = (Cases_popolo−3.44109)/3.21659;

scaled_DosesAdmistratedApril = (DosesAdmistratedApril−3 704 380)/16 144 900;

scaled_Dosesvaccineper100inhabApril = (Dosesvaccineper100inhabApril−14.2995)/19.781;

scaled_DosesAdmistrated15March21 = (DosesAdmistrated15March21−1 631 560)/5 219 560;

scaled_Dosesvaccineper100inhab15March21 = (Dosesvaccineper100inhab15March21−6.96745)/13.6092;

scaled_TotalVaccinated15March21 = (TotalVaccinated15March21−381 143)/827 631;

scaled_L.TotalCases = (L.TotalCases−10.7509)/3.03837;

scaled_L.Cases_popolo = (L.Cases_popolo−4.33326)/3.07371;

scaled_L.DeathsApril = (L.DeathsApril−6.64982)/3.01081;

scaled_L.FatalityRateApril = (L.FatalityRateApril−0.676115)/0.53493;

scaled_L.DosesAdmistratedApril = (L.DosesAdmistratedApril−12.4364)/2.67888;

scaled_L.Dosesvaccineper100inhabApril = (L.Dosesvaccineper100inhabApril−1.82228)/1.34842;

scaled_L.DosesAdmistrated15March21 = (L.DosesAdmistrated15March21−9.35586)/5.58977;

scaled_L.Dosesvaccineper100inhab15March21 = (L.Dosesvaccineper100inhab15March21−1.10013)/1.1876;

scaled_L.TotalVaccinated15March21 = (L.TotalVaccinated15March21−7.15042)/6.02766;

scaled_D.TotalCases = (D.TotalCases−2536.84)/20 038;

scaled_D.Cases_popolo = (D.Cases_popolo−140.452)/340.351;

scaled_D.DeathsApril = (D.DeathsApril−781.714)/5538.99;

scaled_D.FatalityRateApril = (D.FatalityRateApril−0.444522)/2.12994;

scaled_D.DosesAdmistratedApril = (D.DosesAdmistratedApril−127.31)/657.291;

scaled_D.Dosesvaccineper100inhabApril = (D.Dosesvaccineper100inhabApril−3.0988)/10.2835;

scaled_D.DosesAdmistrated15March21 = (D.DosesAdmistrated15March21−56 908)/410 565;

scaled_D.Dosesvaccineper100inhab15March21 = (D.Dosesvaccineper100inhab15March21−1.27779)/3.76081;

1. Final mathematical results

tanh (−1.42065 + (y_1_1 × −3.39404) + (y_1_2 × −4.73707) + (y_1_3 × 8.95406) + (y_1_4 × −3.94323) + (y_1_5 × −6.82661) + (y_1_6 × −1.7329) + (y_1_7 × −1.63566) + (y_1_8 × 3.88688) + (y_1_9 × −2.34527) + (y_1_10 × 2.33409) + (y_1_11 × −6.0983) + (y_1_12 × −1.45215));

y_2_2 = tanh (−2.14609 + (y_1_1 × 0.136623) + (y_1_2 × 2.73299) + (y_1_3 × −3.49886) + (y_1_4 × 2.21296) + (y_1_5 × −1.13925) + (y_1_6 × 2.72466) + (y_1_7 × 2.44443) + (y_1_8 × 3.7965) + (y_1_9 × 4.03604) + (y_1_10 × 2.07138) + (y_1_11 × 4.26635) + (y_1_12 × −2.61202));

y_2_3 = tanh (0.571144 + (y_1_1 × 0.885384) + (y_1_2 × 8.44042) + (y_1_3 × 5.2873) + (y_1_4 × −1.76974) + (y_1_5 × 0.706614) + (y_1_6 × 5.89512) + (y_1_7 × −1.40191) + (y_1_8 × −6.25772) + (y_1_9 × −5.32905) + (y_1_10 × −2.06345) + (y_1_11 × −4.01104) + (y_1_12 × −1.31832));

y_2_4 = tanh (1.47304 + (y_1_1 × −9.41355) + (y_1_2 × −3.54629) + (y_1_3 × 0.245785) + (y_1_4 × 5.5926) + (y_1_5 × −8.12707) + (y_1_6 × 4.30913) + (y_1_7 × 5.65796) + (y_1_8 × 1.87443) + (y_1_9 × 5.30923) + (y_1_10 × −1.79709) + (y_1_11 × 3.23201) + (y_1_12 × 0.0419988));

y_2_5 = tanh (3.10152 + (y_1_1 × −2.23974) + (y_1_2 × −1.37414) + (y_1_3 × −2.56631) + (y_1_4 × 0.420232) + (y_1_5 × 2.6237) + (y_1_6 × −2.10451) + (y_1_7 × 4.5227) + (y_1_8 × −8.65126) + (y_1_9 × 1.78936) + (y_1_10 × −3.66684) + (y_1_11 × 2.90734) + (y_1_12 × 7.43724));

y_2_6 = tanh (0.560944 + (y_1_1 × −5.57786) + (y_1_2 × 0.986413) + (y_1_3 × −0.738111) + (y_1_4 × −1.95099) + (y_1_5 × 0.829621) + (y_1_6 × 1.45241) + (y_1_7 × −2.89629) + (y_1_8 × 2.03976) + (y_1_9 × 5.16157) + (y_1_10 × −1.60565) + (y_1_11 × −0.814266) + (y_1_12 × −4.6738));

y_2_7 = tanh (0.594457 + (y_1_1 × 5.05969) + (y_1_2 × −8.16641) + (y_1_3 × −0.835004) + (y_1_4 × 5.93846) + (y_1_5 × −2.92998) + (y_1_6 × 0.472404) + (y_1_7 × −4.16513) + (y_1_8 × −3.71026) + (y_1_9 × −1.05414) + (y_1_10 × 2.50229) + (y_1_11 × −1.9919) + (y_1_12 × 2.71732));

y_2_8 = tanh (4.30013 + (y_1_1 × −5.42082) + (y_1_2 × −1.7049) + (y_1_3 × 3.86285) + (y_1_4 × −3.49551) + (y_1_5 × −1.38505) + (y_1_6 × 5.31073) + (y_1_7 × 1.28879) + (y_1_8 × 1.53534) + (y_1_9 × 2.92565) + (y_1_10 × −4.6453) + (y_1_11 × −4.55057) + (y_1_12 × −3.19376));

y_2_9 = tanh (−3.39936 + (y_1_1 × −2.78159) + (y_1_2 × −7.09474) + (y_1_3 × 2.17625) + (y_1_4 × 1.32365) + (y_1_5 × −6.56213) + (y_1_6 × 2.69674) + (y_1_7 × 4.87133) + (y_1_8 × −4.79233) + (y_1_9 × 2.97694) + (y_1_10 × 2.77616) + (y_1_11 × 0.397988) + (y_1_12 × −0.136498));

y_2_10 = tanh (−2.32782 + (y_1_1 × −0.98874) + (y_1_2 × 5.12131) + (y_1_3 × 2.8198) + (y_1_4 × 7.51709) + (y_1_5 × −4.07128) + (y_1_6 × 2.71003) + (y_1_7 × −2.83528) + (y_1_8 × −4.85167) + (y_1_9 × −0.409918) + (y_1_10 × 3.68647) + (y_1_11 × 3.31492) + (y_1_12 × 2.97374));

y_3_1 = tanh (1.39119 + (y_2_1 × 7.12212) + (y_2_2 × 2.54375) + (y_2_3 × 2.7966) + (y_2_4 × 0.125616) + (y_2_5 × −4.1964) + (y_2_6 × −4.77784) + (y_2_7 × −6.34753) + (y_2_8 × −0.0837408) + (y_2_9 × 6.11543) + (y_2_10 × −5.83674));

y_3_2 = tanh (−1.504 + (y_2_1 × −7.85682) + (y_2_2 × −6.53737) + (y_2_3 × 0.748975) + (y_2_4 × 2.07492) + (y_2_5 × −1.22556) + (y_2_6 × 1.97351) + (y_2_7 × −0.89566) + (y_2_8 × −0.367512) + (y_2_9 × 3.88914) + (y_2_10 × 5.25445));

y_3_3 = tanh (4.25206 + (y_2_1 × 2.25461) + (y_2_2 × 3.32096) + (y_2_3 × 5.48112) + (y_2_4 × −0.294713) + (y_2_5 × 4.69679) + (y_2_6 × −3.03318) + (y_2_7 × 3.72398) + (y_2_8 × 4.99578) + (y_2_9 × 3.27348) + (y_2_10 × −0.24331));

y_3_4 = tanh (5.56335 + (y_2_1 × −4.57575) + (y_2_2 × −5.57908) + (y_2_3 × −8.18809) + (y_2_4 × 7.38807) + (y_2_5 × 1.92916) + (y_2_6 × 5.85921) + (y_2_7 × −6.42909) + (y_2_8 × 1.03946) + (y_2_9 × −2.60374) + (y_2_10 × 10.4997));

y_3_5 = tanh (−0.804268 + (y_2_1 × 4.45784) + (y_2_2 × 1.03842) + (y_2_3 × −1.59496) + (y_2_4 × 12.9812) + (y_2_5 × −4.6989) + (y_2_6 × 6.61949) + (y_2_7 × −5.95164) + (y_2_8 × 4.69671) + (y_2_9 × 5.57528) + (y_2_10 × −4.6567));

y_3_6 = tanh (−7.42 + (y_2_1 × −4.68621) + (y_2_2 × −4.68574) + (y_2_3 × −3.45686) + (y_2_4 × 3.90072) + (y_2_5 × −9.04524) + (y_2_6 × −1.83576) + (y_2_7 × 3.69017) + (y_2_8 × 7.30282) + (y_2_9 × −5.94748) + (y_2_10 × −0.125969));

y_3_7 = tanh (4.13143 + (y_2_1 × −1.70416) + (y_2_2 × −3.93817) + (y_2_3 × 6.44481) + (y_2_4 × −3.57214) + (y_2_5 × 3.52104) + (y_2_6 × −5.98477) + (y_2_7 × −3.52718) + (y_2_8 × −5.20738) + (y_2_9 × 4.03708) + (y_2_10 × 2.49501));

y_4_1 = tanh (5.59629 + (y_3_1 × −0.169289) + (y_3_2 × 4.72624) + (y_3_3 × −5.26795) + (y_3_4 × 4.30637) + (y_3_5 × −4.98808) + (y_3_6 × 2.80358) + (y_3_7 × 1.47525));

y_4_2 = tanh (−4.56394 + (y_3_1 × 11.2667) + (y_3_2 × 3.36337) + (y_3_3 × −7.04849) + (y_3_4 × −11.4603) + (y_3_5 × 4.59576) + (y_3_6 × −9.33503) + (y_3_7 × 5.08033));

y_4_3 = tanh (6.95199 + (y_3_1 × −6.86011) + (y_3_2 × −4.36792) + (y_3_3 × 3.40104) + (y_3_4 × −11.7852) + (y_3_5 × 12.1614) + (y_3_6 × 4.34408) + (y_3_7 × −13.2492));

y_4_4 = tanh (5.01676 + (y_3_1 × −3.78135) + (y_3_2 × −5.67526) + (y_3_3 × 2.34227) + (y_3_4 × −8.2123) + (y_3_5 × 4.20318) + (y_3_6 × −14.1666) + (y_3_7 × 2.23312));

y_4_5 = tanh (−6.53588 + (y_3_1 × −5.03423) + (y_3_2 × −5.34327) + (y_3_3 × −5.58299) + (y_3_4 × −4.09033) + (y_3_5 × −5.42264) + (y_3_6 × −1.05588) + (y_3_7 × −1.83719));

y_5_1 = tanh (1.84566 + (y_4_1 × −2.5998) + (y_4_2 × 0.0872705) + (y_4_3 × −3.38745) + (y_4_4 × 12.681) + (y_4_5 × −5.77164));

y_5_2 = tanh (1.82072 + (y_4_1 × −3.00937) + (y_4_2 × −9.32762) + (y_4_3 × −1.06101) + (y_4_4 × −3.57497) + (y_4_5 × 5.42249));

y_5_3 = tanh (0.929102 + (y_4_1 × −7.35493) + (y_4_2 × −11.5461) + (y_4_3 × 9.13327) + (y_4_4 × 3.11303) + (y_4_5 × 5.28057));

y_5_4 = tanh (−0.327379 + (y_4_1 × −10.9707) + (y_4_2 × −7.26578) + (y_4_3 × 17.5456) + (y_4_4 × 2.52181) + (y_4_5 × −6.04749));

y_5_5 = tanh (−5.19209 + (y_4_1 × 6.30455) + (y_4_2 × 2.20143) + (y_4_3 × −0.381196) + (y_4_4 × −19.6165) + (y_4_5 × −0.986056));

y_5_6 = tanh (1.52476 + (y_4_1 × −3.5201) + (y_4_2 × 7.39273) + (y_4_3 × −9.6574) + (y_4_4 × −1.32885) + (y_4_5 × 3.68376));

y_5_7 = tanh (−0.0014946 + (y_4_1 × −0.783583) + (y_4_2 × −5.31838) + (y_4_3 × −2.04202) + (y_4_4 × −5.78655) + (y_4_5 × 0.0888508));

scaled_DeathsApril = (−0.789209 + (y_5_1 × −10.5257) + (y_5_2 × −0.139852) + (y_5_3 × −0.508132) + (y_5_4 × 1.26922) + (y_5_5 × −11.2899) + (y_5_6 × 0.723576) + (y_5_7 × 0.952592));

scaled_FatalityRateApril = (−0.874703 + (y_5_1 × 5.79615) + (y_5_2 × −0.0328672) + (y_5_3 × −7.27996) + (y_5_4 × 13.8602) + (y_5_5 × −2.39408) + (y_5_6 × 6.62376) + (y_5_7 × 8.21696));

(DeathsApril, FatalityRateApril) = (0.5 × (scaled_DeathsApril + 1.0) × (386 416-1) + 1,0.5 × (scaled_FatalityRateApril + 1.0) × (25-1) + 1).

1. List of countries

**Table:**

Afghanistan	Ghana	Oman
Albania	Greece	Pakistan
Algeria	Grenada	Palestine
Andorra	Guatemala	Panama
Angola	Guinea	Papua New Guinea
Antigua and Barbuda	Guinea-Bissau	Paraguay
Argentina	Guyana	Peru
Armenia	Haiti	Philippines
Australia	Honduras	Poland
Austria	Hungary	Portugal
Azerbaijan	Iceland	Qatar
Bahamas	India	Romania
Bahrein	Indonesia	Russia
Bangladesh	Iran	Rwanda
Barbados	Iraq	Saint Kitts and Nevis
Belgium	Ireland	Saint Lucia
Belize	Israel	Saint Vincent and Grenadine
Benin	Italy	Salomon Islands
Bhutan	Ivory Coast	Samoa
Bolivia	Jamaica	San Marino
Bosnia Herzegovina	Japan	Sao Tome and Principe
Botswana	Jordan	Saudi Arabia
Brazil	Kazakhstan	Senegal
Brunei	Kenya	Serbia
Bulgaria	Kirghizstan	Seychelles
Burkina Faso	Kosovo	Sierra Leone
Burundi	Kuwait	Singapore
Byelorussia	Laos	Slovakia
Cambodia	Latvia	Slovenia
Cameroon	Lebanon	Somalia
Canada	Lesotho	South Africa
Cape Verde	Liberia	South Korea
Central African Republic	Libya	South Sudan
Chad	Liechtenstein	Spain
Chile	Lithuania	Sri Lanka
China	Luxembourg	Sudan
Colombia	Madagascar	Suriname
Comoros	Malawi	Swaziland
Congo	Malaysia	Sweden
Costa Rica	Maldives	Switzerland
Croatia	Mali	Syria
Cuba	Malta	Taiwan
Cyprus	Marshall Islands	Tajikistan
Czech Republic	Mauritania	Tanzania
Democratic Republic of Congo	Mauritius	Thailand
Denmark	Mexico	Timor Est
Diamond Princess	Micronesia	Togo
Djibouti	Moldavia	Trinidad and Tobago
Dominica	Monaco	Tunisia
Dominican Republic	Mongolia	Turkey
Ecuador	Montenegro	Uganda
Egypt	Morocco	Ukraine
El Salvador	Mozambique	United Arab Emirates
Equatorial Guinea	MS Zaandam	United Kingdom
Eritrea	Myanmar	United States
Estonia	Namibia	Uruguay
Ethiopia	Nepal	Uzbekistan
Fiji	Netherlands	Vanuatu
Finland	New Zealand	Vatican City
France	Nicaragua	Venezuela
Gabon	Niger	Vietnam
Gambia	Nigeria	Yemen
Georgia	North Macedonia	Zambia
Germany	Norway	Zimbabwe
